# Obstetric violence and disability overlaps: obstetric violence during child birth among womens with disabilities: a qualitative study

**DOI:** 10.1186/s12905-022-01883-y

**Published:** 2022-07-18

**Authors:** Aregahegn Wudneh, Aneleay Cherinet, Mesfin Abebe, Yesuneh Bayisa, Nebiyu Mengistu, Wondwosen Molla

**Affiliations:** 1 Department of Midwifery, Dilla University, PO. Box (DU): 419, Dilla, Ethiopia; 2 Department of Psychiatry, Dilla University, P.O. Box (DU): 419, Dilla, Ethiopia; 3 School of Medicine, Dilla University, P.O. Box (DU): 419, Dilla, Ethiopia

**Keywords:** Women, Obstetric violence, Ethiopia

## Abstract

**Introduction:**

Obstetric violence is an invisible wound which is being distorting the quality of obstetric care. Obstetric Violence, which is an issue spoken and amplified currently as a type of sexual violence and is of alarming seriousness and is an evolving field of inquiry despite women’s experience of institutional childbirth, has garnered unprecedented global attention in recent years. Losing on both counts: obstetric violence is a double burden among disabled women.

**Aim:**

To explore the experience of disabled women towards obstetric violence during child birth in Gedio zone, South Ethiopia.

**Methods:**

Twenty-two (22) women with disabilities were interviewed. They were recruited through a nonprobability snowball sampling method. The interviews were conducted using a structured questionnaire in the Gedio zone, south Ethiopia. For coding purposes, NVivo (version 11) software was employed. Using a method known as continuous comparison, we classified the extracted codes based on their similarities and differences. The classes were then arranged in such a way that there was the greatest internal uniformity and the least external mismatch.

**Results:**

The profile of the study group is predominantly of women between the ages of 21 and 30. Physical abuse, verbal abuse, stigma and discrimination, neglect and abandonment, and violations of privacy were the five major categories emerged during the thematic analysis describing the experience of obstetric violence. Women also observed these forms of obstetric violence among other disabled women during child birth. In addition to the violations of care, some of the participants described positive aspects of their childbirth experiences in one or more obstetric care settings.

**Conclusion:**

This study concluded that the quality of service was deplorable, with reports of obstetric violence among this vulnerable group of women imposing a double burden on them. The findings suggest that there is a need to improve maternity care for disabled women by implementing comprehensive, culturally sensitive, client-sensitive special services and providing sensitivity training to healthcare providers, ensuring satisfied, equitable, and quality obstetric care.

## Introduction

The term “disability” is well defined as “lasting physical, intellectual, or sensory impairment that, when joined with other blockades, may avert them from participating wholly and successfully within a society on an equal basis with others.” [1].

There is no commonly recognized definition of disability. The International Classification of Functioning, Disability, and Health states disability as “covering impairments, activity restrictions, and participation limitations,” and adds that “disability is a circumstantial variable, dynamic over time and in relation to context. As a result, disability is considered a "multifaceted spectacle reflecting an dealing between the characteristics of a person's body and features of the society in which he or she lives [2].

According to the World Report on Disability, published jointly by the World Bank and WHO in 2011, Ethiopia had 15 million people with disabilities, accounting for 17.6 percent of the total population at the time. According to the Ministry of Labour and Social Affairs, 95 percent of people with disabilities in the country are poor, with the vast majority living in rural areas where basic services are scarce and access to rehabilitative or support services is difficult [3, 4].

Obstetric violence is an unseen wound that distorts obstetric care quality. Obstetric violence is a serious issue that is currently being discussed and amplified as a type of sexual violence. Despite the fact that a woman's experience of institutional childbirth has received unprecedented global attention in recent years, it is still an evolving field of study [5–7].

A human rights issue, obstetric violation, to coin a phrase, is a pressing legal problem around the globe. It violates women's "right to a life free of violence [8]." Obstetric violence is a type of sexual violence that is currently being discussed and highlighted as a major issue. Despite the fact that a woman's experience of institutional childbirth has received unprecedented global attention in recent years, it is a developing field of study [9, 10]. Obstetric violence as a breach Human right is worser among disabled women despite of all disabled persons are right holders and that impairment may not be used as a justification for denial or restrictions of human rights [7, 11].

OV is addressed in the WHO declaration on obstetric violence as it not only violates women's rights to respectful care, but it can also jeopardize their, rights to life (as a human right violation), health, bodily integrity, and freedom from discrimination [12–14].

Having child women all over the world are experiencing obstetric violations (part of a human rights violation) in various forms, such as dehumanized assistance, abuse of interventionist actions, medicalization, sexual violence, and reversion of the process from natural to pathological, resulting in a loss of their right to decide about their own body and a negative impact on the quality of women's lives, which has a negative impact on the quality of maternity care [15–17].

Obstetric violence is more prevalent in women who are defenseless, vulnerable, and powerless, and who are subjected to more severe and cruel violent and hostile practices during pregnancy and childbirth: disabled women. From this vantage point, we understand that the elderly, as well as children, adolescents, and people with disabilities, are circumstantially affected and vulnerable; that is, they are “violated,” and they demand the application of norms that recognize materially reproduced inequality in order to maintain their dignity [18–20].

Many researchers believe that women feel disempowered in the non-medicalized midwifery childbirth model. While this disempowerment is not always the result of violence, many women experience profound incompetence, a burden on healthcare providers, frustration, and shame as a result of "failing" to give birth without medical assistance [21, 22].

A qualitative study also discovered that childbirth care was unsettling for women, particularly when providers treated us as objects [23, 24] Obstetric violence against disabled women becomes even more “normalized” and institutionalized than violence against nondisabled women. The story of a deaf parturient who, despite knowing she was pregnant, had no idea she was carrying twins was told. The second child died shortly after the first child was born because medical personnel were unable to connect with the mother due to their complete ignorance and lack of preparation [25].

Women who were deaf were sterilized against their will. To meet sterilization targets or quotas, health professionals performed cesarean sections for profit sterilizing patients using the public service, by tubal ligation or withdrawal of the uterus, without the patients' knowledge. [9, 26, 27].

Ample of research’s evidenced institutional delivery, nowadays, there is a paradigm shift away from promoting institutional delivery and toward emphasizing quality, violence-free obstetric care for every woman regardless of their background. Providing universal access to safe, acceptable, and high-quality sexual and reproductive health care has the potential to significantly reduce global rates of maternal morbidity and mortality [14, 28].

OV is spreading around the world, causing child-bearing women to suffer from obstetric violence rather than labor pain. It is more common in developing countries, including Ethiopia, and worse among vulnerable groups, such as disabled women. A negative maternity experience may significantly increase the risk of negative health outcomes for the mother with disability. Obstetric violence for such type mother become the extra burden [14, 19].

In Ethiopia, as per the researcher knowledge, there is no research on obstetric violence among disabled women has been conducted. On the other hand, WHO urges all health care providers to pay attention to this invisible but deep wound and calls for researchers to investigate. Means that governments must ensure that the human rights implicated by obstetric violence are respected within public sector health facilities, but also that women are protected from abuse when they seek care in the private sector. In existing norms, which are also out of date in terms of prevention and instruments capable of accommodating victims, the situation of women with disabilities remains virtually invisible, particularly in developing countries such as Ethiopia. In addition, due to the fact that the concept itself and the precise characterization of people with disabilities are still under construction and because "their voices are always silenced, nullified by the weight of the multiple problems they suffer, obstetric violence could further make the situation of women with disabilities more challenging. This study may be more useful in revealing this invisible wound among the most overlooked population by demonstrating how much worse the problem is among disabled women. Furthermore, it benefits survivors of obstetric violence by providing access to effective solutions. As a result, the first national study sought to investigate disabled women's experiences of obstetric violence during childbirth in South Ethiopia's Gedio zone.

## Methods and materials

### Study setting

This research was carried out in Ethiopia's Gedeo Zone, in the country's southernmost region. Dilla is the zone’s administrative center. It is 345 km from Addis Ababa, Ethiopia's capital city. This zone has a total population of 847,434 people, of whom 422,692 are women and 424,74 are men according to the 2007 census conducted by Ethiopian Central Statistical Agency. The majority of the population was Protestant, with 73.21 percent identifying as such, while 10.67 percent identified as Orthodox, 7.96 percent as traditional religion, 2.44 percent as Muslim, and 2.11 percent as Catholic. There is one referral hospital and three primary hospitals in the zone that provide maternity services.

### Approach

From February 10/2022 to April 10/2022, the research was carried out in Gedeo Zone, South Ethiopia. Because little is known about obstetric violence among disabled women, a qualitative, descriptive, phenomenological study design with in-depth interviews was employed. This study used a phenomenological approach to clarify and illuminate how people understand and comprehend obstetric violence in disabled women. Because obstetric violence against disabled women is a complex and hidden issue, the in-depth interviews are designed to elicit a more detailed response.

### Participant’s recruitment

Women with disabilities who had previous experience with institutional delivery and had lived in the Gedeo zone for at least six months were included. The study participants were sampled using purposive sampling, and the participants were found using the exponential discriminative snowball sampling technique. Each participant received multiple referrals; however, each referral resulted in the recruitment of only one participant. We also used field assistants to locate participants in need and approach study participants through them.

### Inclusion/exclusion criteria

The current study included all disabled women who had had at least one previous institutional childbirth. However, disabled women who had not given birth in a facility in the previous 24 months and had not lived in the selected catchment area for at least six months were barred from participating in the current study.

### Data collection tools and procedures

An in-depth interview semi-structured guiding question was used to collect data from 22 women with disabilities. After reviewing the relevant literature, the data collection tool was developed first in English. It was translated to Amharic and Gedio languages and then back translated to English by an independent translator for consistency. Before collecting data, all questions about the study's objectives, risks, goals, and potential risks and benefits were answered. Tape recorders and field notes were used to record interviews with study participants, and data was collected using both the tape recorder and field notes.

All interviews were conducted in Amharic and the local language (Gedeo-Uffa) by male master's degree holders who worked as lecturers and assistant professors at Dilla University, as well as two qualitative study expert supervisors. In-depth interviews were conducted in people's homes, outdoor living areas, and open and closed spaces in a quiet location with no one else present except the interviewees. Each interview lasted 40–50 min on average. As a result, we conducted extensive semi-structured interviews. Following the first interview, we used the snowball sampling method to identify the next participant (s).

The number of participants enrolled was determined by data saturation; that is, data collection was stopped once no new themes were discovered and a theoretical saturation point was achieved. During the data analysis process, all participants were subsequently numbered to aid in organization and analysis. Furthermore, all interviews were audio-recorded and were conducted with a predetermined list of open-ended questions.

During the interviews, data collectors took field notes, including memos of participant behavior and contextual aspects, to ensure that the data was triangulated with the record. In addition, a code was assigned to each participant to be used as a nickname.

### Data handling and analysis

Following the transcription of the data from the interviews, the researchers read the texts several times to create more compact semantic units. For coding purposes, NVivo (version 11) software was employed. We classified the extracted codes according to their similarities and differences by using the continuous comparison method. The classes were then ordered in a way that there was the most internal uniformity and the least external mismatch. Furthermore, the accuracy and precision of the initial coding were checked in a standard procedure overseen by qualified experts in the field. To reduce bias, each coder independently coded the interviews, and differences were discussed to determine the initial coding framework.

Themes and subthemes were reviewed and verified by other researchers to increase the study's validity. Finally, the themes, subthemes, and representative quotations were translated into English by the researchers. Furthermore, quotations were edited to correct grammar or to remove content with repeated words or stutters.

### Strategies conducted to improve quality

Credibility: The investigators' long-term involvement (prolonged engagement) in the gedio zone research setting and participation in much qualitative research has aided in the collection of more data and data from a variety of perspectives. Furthermore, the researchers had formal training in qualitative research, which added credibility to the findings.

Transferability: The data was transcribed using verbatim transcription (golden approach), which ensured rich data and detailed descriptions. As a result, others were able to determine the applicability of the findings to their own situations.

Respondent validation: After transcribing all collected data, we presented it to the study participants and confirmed that it was their response. The participants testified about the discovery.

Peer debriefing: After transcribing all data, one transcriber checked the other transcribers' transcriptions against the recorded audio. Then any discrepancies were ironed out. (Table 1).

**Table 1:** Analytical process of the data

Meaning units (selected)	Themes	Categories
Received unnecessary discomfort or pain relief treatment	Inappropriate car	Physical violence
Laboring women's legs are forcefully spread apart	Lack of empathy	
Laboring women Received care in an uncomfortable, painful position		
Laboring women felt received a painful assist as opposed to labor		
A woman slapped by healthcare providers	Physical force or abrasive behavior	
A woman heated by heath care providers		
A woman Kicked by heath care providers		
A woman pinched by heath care providers		
A care provider shouted in a loud, sharp way	Non-dignified care	Verbal violence
Obstetric care providers mock them or treat them with contempt		
Care providers unusually raised voice		
Support staff insulted women and their companions		
Negative remarks were made to foreshadow negative outcomes	Negative comments	
The obstetric care provider commented on the unnecessity of getting pregnant and having a disability		
Being threatened with the bad outcomes of pregnancy with disability		
feel or declare that being responsible for a fault or wrong	Lack of trust by Obstetric care providers	
Being made to feel guilty about her pregnancy		
The obstetric care provider blamed women for being disabled		
Denied from food or fluids in labor, unless medically necessitated	Lack of right to timely healthcare	Neglect and abandonment
Being rejected by health care providers		
left alone in the absence of a companion or caregiver		
Potential development of harmful thoughts, actions, or emotions	Loss of trust on facility care	
Negative attributions and suspicions towards facility care		
Struggling alone to survive		
Women planned not to give birth at a facility in the future		
Fear of being regarded as repulsive	Not being seen as a human individual	Stigma and Discrimination
Feeling to be unworthy of respect or attention		
Fear of being a burden on others		
Being considered an object upon which physicians can act at will		
Feeling less important or useless		
Being reprimanded by the physician for being disabled		
Being incompetent for childbirth		
Feeling of helplessness during child bearing		
Feelings of receiving partial care	Luck of equitable care	
Women received inadequate obstetric care		
Women received obstetric care regarding their status		
The mother and her newborn locked away in a different room	Isolation	
A woman and her newborn are locked in the restroom		
Feeling Health providers discussed private health information in a way that others could hear	lack of privacy during maternal care	Non confidential care
Being left to be watched and stood by people		
Feeling Everything has deteriorated like windows were broken, doors wide open for everybody to enter without any restriction		
Discussing private or personal issues with the medical team		
Feelings of publicity about private issues		
The providers didn’t use drapes or coverings appropriate to protect the mother’s privacy	Unethical/nonprofessional care	
Being left naked on the delivery coach		

## Result

### Socio-demographic characteristics

The study participants included were 22 mothers with different forms of disability who experienced obstetric violence in a different form. Most were housewives (n = 14), four were government employees, and four were private business runners. The profile of the study group is predominantly of women aged between 21–30 years. More than half of the respondents (n = 13) were from Gedeo ethnic group, most were Christian religion (n = 17) followers. Regarding the marital status of the mothers, the majority(n = 20), were married, nearly all participants were considered to have low- middle income, the majority of the participants were completed elementary school, and the majority lived in A nuclear family (having parents and dependent children) as shown in Table 2.

**Table 2:** Socio demographic characteristics of participants in Gedeo zone, south, Ethiopia, 2020(n = 22)

Variable	Frequency (N = 22)
*Age, years*	
15–20	5
21–30	10
31–40	7
*Occupation*	
House wife	14
Government employee	4
Private business	4
*Education*	
No formal education	4
Read and write	2
Primary and secondary	5
Collage and above	4
*Religion*	
Christian	11
Muslim	4
*Marital status*	
Currently married	20
Divorced	1
Widowed	1
*Family size*	
2–3	8
4–5	9
≥ 6	5
*Ethnicity*	
Gedeo	13
Oromo	3
Amhara	3
Others	3
*Income*	
Less than 2500	15
Greater than 2500	7
*Residence*	
Urban	9
Rural	13

### Obstetric characteristics

Most of the participants in the study group were multiparous (n = 20). Regarding obstetric complications during pregnancy and child birth, the majority had no bad obstetric history (n = 17), followed by a history of abortion and intrauterine fetal death (n = 3) and (n = 2) respectively. Majority (n = 15) had at least one antenatal visit during a recent pregnancy, as seen in Table 3.

**Table 3:** Obstetric characteristics of the respondents

Variables	Frequency	Percentage
*Number of pregnancies*		
1–2	9	40.9
≥3	13	59.1
*Number of children*		
0	2	9.1
1–2	8	36.4
≥3	12	54.5
*ANC visit*		
Yes	15	68.2
No	7	31.8
*Abortion*		
Yes	3	13.6
No	19	86.4
*Still birth*		
Yes	2	9.1
No	20	90.9
*Number of facility birth*		
1–2	16	72.7
≥3	6	27.3

The WHO disability assessment tool was used to assess and observe respondents for their disability status. Accordingly, Physical (long-standing physical condition and long-standing), sensory (deafness or severe hearing impairment), sensory (blindness or partial sightedness), and having a combination of two or more of these were the four disability types identified among the participants. Finally, we discovered that the majority of participants (n = 10) had physical or long-standing physical conditions. Then there are visual impairments (n = 8), as shown in Table 4.

**Table 4:** Participants’ disability status

S.no	Pseudonym	Disability status
1	W4, W5, W3, W7, W9, W12, W16, W18, W20 and W22	Physical (long-standing physical condition)
2	W2, W8, W10, W13, W15, W17, W11 and W21	Sensory (and blindness or partial sightedness)
3	W14 and W6	Sensory (deafness or severe hearing impairment)
4	W1 and W19	Having a combination of two or more disabilities

### Context of obstetric violence among the participants

The analytical process revealed five major categories describing the experience of obstetric violence: physical abuse, verbal abuse, stigma and discrimination, neglect and abandonment, and violations of privacy.

In addition to the violations of care, some of the participants described positive aspects of their childbirth experiences in one or more obstetric care settings, including immediate postnatal care. Women spontaneously brought up the topic of obstetric violence, illustrating the negative experiences they had faced, witnessed, or heard about from others, where they were “well cared for” by the “helping hand” of healthcare providers.

The majority of the participants described both scenarios in which they felt they had been subjected to obstetric violence and those in which they witnessed a colleague commit violent acts against a specific woman. Women offered explanations for why this situation occurred, and the majority of participants saw them as intentional violence of care due to their disability. However, some of them also stated that obstetric violence is explained by an overburdened health system, rather than isolated incidents of intentional abuse of care. A woman with a physical disability living in an urban residence, for example, explained that what women perceive as neglect or abandonment by a healthcare provider may actually be the result of an understaffed facility:

*…” If a care provider is already delivering my baby and another woman calls for his attention, you can bet he will not be able to attend to her at that time. Is not that correct? No, but that the patient may believe she has been treated unfairly. Is not that right? However, we all know that is not the case. Despite the obstetric care provider's commitment to assisting me in having a healthy baby, she may feel neglected. Of course, because of my disability, I may require more attention than others. With these issues, I could not blame healthcare providers for committing obstetric violations on purpose (women 1, 29 years old, urban women).*

### Category-one

#### Physical abuse

Women described detailed scenarios in which women were slapped, pinched, forcefully, wide opened their legs until they felt severe pain or beaten during childbirth, and it was widely assumed that slapping and other types of physical abuse were used to ensure the woman's and the baby's health among the laboring mothers. Furthermore, if a woman closes her legs during childbirth, health workers may slap her or forcefully open her legs wide to "encourage" or "give her strength" to "open up and deliver well." When given the freedom to move, women may deliver their babies in a variety of positions, including squatting or lying on their sides, which is often more comfortable for mothers with physical disabilities to their back. This position may also aid in the positioning of the baby. Regardless of their physical condition, some women report being forced onto their backs and restrained in a supine position during the final stages of pushing. This type of violence may be worse among disabled women due to their uncooperative nature of their disability status.

*“…Of course, my leg condition was preventing me from cooperating. The midwife instructed me to spread my legs wide apart. You understand that the baby's head is out, so you must cooperate with me by widening your legs by slapping my thighs. She was attempting to deliver the remainder of my baby's body. The midwife who was delivering the baby at the time was agitated. As a result, I do not believe she was indirectly assisting me with my baby because of obstetric violence. (Women1, 36 years old, rural women).*

Slapping is also used to gain a woman's compliance and cooperation and is frequently not regarded as obstetric violence by women as long as it is not done with the desire to harm. It is common to overcome the challenges that care providers may face in patient operations during busy staffing and high admission rate occasions by engaging patients in cooperation. Again, the problem may be a large burden for disabled women as they prefer to slap over bud pregnancy outcomes.

*“…. I felt bad, but when I delivered the baby, I realized they were assisting me despite my condition, which prevented me from cooperating with them. I did not think about it and went again, because if that baby dies, I am out. If I perish, we all perish. Therefore, I had rather have that slap than miss the baby”. (Women1, 36 years old, rural women).*

*“…. I was in labor for nearly five hours at home. I went to the health center after the labor became more intense. As soon as I arrived at the health center, the nurse checked on me and my baby. I lied on my right side during the exam because I could not be on my back due to severe swelling in my lower back since childhood. The nurse told me to get on my back after 2 h of labor at the health center. However, I replied that I could not be on my back because of my swelling. Please deliver my baby in another comfortable position, and he yelled back, "Get on your back now, or you will hug a dead baby”. Then I had no choice but to lie on my back to save my baby. (Women18, 29 years old, urban woman).*

### Category-two

#### Neglect and abandonment

Women frequently felt abandoned during labor and were unable to summon obstetric care providers when they were required. They were rarely monitored during labor, and if complications arose, such as excessive bleeding, it was difficult to get an obstetric care provider's attention. Deaf women confirmed that they felt overworked in some cases and did not take the necessary time to address the woman's needs. During labor and delivery, communication barriers were also present, especially for women of color. The situation was exacerbated for these women. All deaf participants stated that they did not believe they and the staff were communicating effectively. A woman also described her facility delivery with vision impairment as she left the toilet room with her newborn and mother after a spontaneous vaginal delivery. This is not only a reflection of obstetric violence, but also a series of human rights violations. Women stated that they were unable to communicate their pain as a result of being left alone on the toilet with their mothers and newborns, as well as their needs. This type of violation may result in a number of complications for both the newborn and the mother, such as infection from a polluted environment or preventable fetal or neonatal death if not treated promptly.

*… “I was having trouble. I was in labor for three hours. It was a difficult task. Furthermore, I had the impression that the care provider was speaking to me while I was in labor, but I could not understand due to my hearing impairment. He eventually left me alone in the room. In the interim, another caregiver arrived and cared for another woman. I felt bad for my baby and his condition because no one was there to inform me of his condition. Finally, I decided to go outside and ask my family to assist me by requesting care providers to check on my progress, but they would not let me and did not understand my concerns. After all, a caregiver rushed to me after I had been missing for more than two hours." (Women 6, 30 years old, urban facility).*

*… “I was in excruciating labor. It was my very first child. While I was in labor, the midwife who was assisting me was busy tending to other mothers in labor. He had left me alone for over 30 min. After all, he showed up, and I gave them my phone number for an interpreter and told them to bring me some drinks because I was thirsty, but they never called. As a result, I keep the feeling to myself. (Women 14, 25 years old, rural women).*

*… “My most recent child was born in a health center. As you can see, I am blind. I went blind at a young age. I went to the health center with my mother to receive maternity care from obstetric care providers. When I arrived at the facility, the caregivers placed me on a bed and checked my labor status. My mother remained in the delivery room with me until they told her to leave. I gave birth successfully shortly after my mother left the delivery room, thanks to the excellent care of the midwives. They transferred me to another room with my little baby after a few minutes of successful vaginal delivery, and I thanked the midwives for the favor they did for me. However, the room they transferred us to was a toilet, as I told you. Me and my baby stayed alone without any care. I was bleeding, my baby was crying, and I was thinking of attempting breastfeeding, but I couldn’t know when to start it. I shouted for help, but still no one came to me except my mother. You know, I forgot the place they put me because my concern was my baby’s condition and the blood I was losing. I really felt bad. I even started to blame my mother, who brought me to that health center with cruel nurses (Women 11, 35 years old, urban women).*

### Category-three

#### Stigma and discrimination

Because of their disability status, the participants expressed fear of discrimination and stigma. To avoid this type of obstetric violence, they even prefer home births over hospital births. Some participants stated that unless there were life-threatening complications, they would not give birth in a facility. Women and their babies may die as a result of home delivery and its consequences if appropriate care is not provided without discrimination or stigma. Women with vision impairments experienced discrimination as well. This type of obstetric violence, which was completely unhuman, affected both the mother and her baby.

*… “It was a truly revolting scenario. They immediately transferred me and my baby to another separate room after a successful vaginal delivery. I remained in that room until my mother approached me crying, and informed me that the separate room was not a caring room, but rather a filthy toilet. I was furious at the time, especially for my unfortunately 1-h-old baby, who was forced to stay at the toilet without his sins. "Why did they decide to put me and my baby there?" I always wonder. Is it because I am blind? Allow it to be, but why the baby? Is he not like the other babies? This, you see, is more painful than the labor itself. …” I was in a terrible mood. I kept returning to the situation in my mind. I tried to convince myself that it was not intentional and that it was possibly due to the overcrowding in the postnatal rooms. At the same time, I question myself, asking why me and not others. Is it because I cannot see where they have placed me? Finally, I regret having my baby at that hospital”. (Women 11, 35 years old, urban women).*

### Category-four

#### Non- confidential care

Obstetric violence was exacerbated by the facility's structure, as some women felt their privacy was violated by the poor design of the labor wards, where women would be exposed to other patients, their families, and providers. There were no partitions between the beds in the delivery room, and if curtains were present, they were tattered or not properly closed. The windows were shattered, and there were no curtains to shield the women from onlookers. Therefore, many people are watching the delivery process while the woman is receiving care naked. The problem was even worse for disabled women.

*…” Even while I was delivering, many people, including those who were not wearing gowns, were passing by and staring at me. While I was laboring on the couch naked, some of them stood and watched. It’s supposed to be enclosed, but they didn't repair everything they said, like the windows; everything has deteriorated, and they did not do anything about it; they did not repair… That is, it is not permitted by our religion. Everyone was staring at us. Because I was having labor pains, I did not say anything.” (Women 4, 28 years old, urban women).*

### Category-five

#### Verbal abuse

Women described shouting, yelling, insults, negative comments, and derogatory remarks from healthcare providers. Some of them had also witnessed such harassment of working women. They described their experiences as demeaning and stated that verbal abuse occurred throughout their stay in the health facility, beginning with their initial contact with healthcare providers and continuing through labor, childbirth, and discharge. The verbal abuse they received was also related to their decision to become pregnant, having a disability, and future competencies for childbirth. Some of the participants also witnessed a woman in labor being treated as unhuman being or animal by obstetric care providers.

*…” During my labor, the midwife who was assisting me frequently shouted and yelled at me. During a vaginal examination, he would say, "open your legs wide enough." Because of my condition and the uncontrollable state of my legs, he was furious with me. I saw the nurse who was caring for other women with hearing impairments insult them. She might not have heard what was said, but I did, and I cried. I gave birth at home because what I saw and felt was more painful than the labor itself. (Women 4, 28 years old, urban women).*

*…” I arrived at the delivery room in a wheelchair. I was 24 years old, a mother of a young child, and expecting twins. Because one of my babies was breech, they said I could not give birth vaginally and that I needed a cesarean section. Shortly after a nurse begins to place surgical equipment behind my head, the majority of my body begins to numb. A male surgeon enters the operating room and inquiries about the number of children I have. "No one," I replied. The surgeon asks me flatly if I want to be tied this time. When I said no, the surgeon looked at me and said, "You must be tied because of your disability condition?” (Women 17, 24 years old, rural women).*

*…you know, it is very painful to be treated as a dog or cow or goat? Isn’t it? Some can be very, very rude. The way they talk to women sometimes as if they are not being sensitive to your situation. You understand? They used to insult a woman in seeking assist like ehn. “Go out you goat who told you to enter this room”? “You see this animal how she behaves”? Sometimes you see mothers blinking their faces and they will be crying. You understand? They Make you feel less important or useless at all because you’ve come to them in need of their help.?” (Women 21, 34 years old, urban women).*

## Discussion

This study explored disabled women’s experiences of obstetric violence during childbirth in the south region of Ethiopia, and provides the first known qualitative evidence of obstetric violence among disabled women during childbirth in Ethiopia, the findings suggest that across urban and peri-urban/rural settings, age groups and religions, disabled women experience acknowledge obstetric violence during childbirth.

Women reported physical abuse such as slapping, stigma, and discrimination such as being locked in the toilet, verbal abuse such as shouting at, intimidating, and negative comments such as being told they are incompetent to be mothers in the future, threatening women with physical abuse, and nonconfidential care such as being left unattended to be observed by many people around them.

Physical abuse was the most common type of obstetric violence in the current study, as almost all participants had experienced this type of obstetric violence during institutional child birth. Physical abuse, as a form of painful forced widening of the legs during labor, was a more painful act than the labor pain itself, according to a woman with long-standing physical problems. Furthermore, the study participants also explained other forms of physical abuse such as slapping, pinching. This suggests that women with this type of disability experience multiple pain overlaps during labor and delivery, including gender, disability, and obstetric violence overlap. It may have a large effect on this type of women health. Primarily, they feel not to receive any maternity care at the health facility, putting them in danger of maternal morbidity and mortality. *This explanation parallels other literature in the area *([19, 29, 30]*.* The same experience was also there in India where the sister (nurse) gave a laboring woman two tight slaps across her face [31]*.*

Based on reports mainly from different parts of the world, particularly from low-income countries, disabled child bearing women experienced physical abuse.

In this study, disabled women also experienced verbal abuse during child birth. They have been experiencing negative comments about their physical status. The women in this study suffered from pain and abusive care than labor pain. The obstetric care providers blamed a woman for being pregnant with disability as the women had no parenting abilities and fear that they will harm their child. This may result in a disabled woman being prevented from becoming a parent, as was the case in Iceland, where women with intellectual disabilities were prevented from becoming pregnant through sterilization and sex-segregated institutions were used to prevent people with this from becoming parents [7]. Similar experience from the study among Deaf Women from Western Cape of South Africa who had experienced a different form of verbal abuse such as they have been yelled by health care providers having bad habits. Nigerian study also explained that women suffered of being insult by obstetric care providers [7, 29, 30]. The study in India also showed the birth experience of women as the nurses committed verbal abuse by shouting at the laboring women [31].

Disabled women in this study experienced discrimination and stigma. Women reported that they were stigmatized just because of their impairments. They were forcefully isolated and taken to a disgusting toilet after the third stage of labor was managed. This is unhuman care to let a woman with her baby to stay at the toilet despite the right of disabled women to receive similar care as nondisabled women. The primary concern with this type of violent obstetric care is the possibility of physical harm to two dependent clients (a disabled woman and her beloved new born) as a result of a fall, as well as improper positioning on the bed, which could be painful and necessitate the presence of a care provider. This is also not only a violation of their right of equity care but also unhuman care letting a mother and their neonate to infection and finally may be to death [32, 33]. Even though the degree of discrimination was less, a similar experience was also there in England where significantly fewer women with disabilities were able to choose a comfortable position most of the time during labour compared with nondisabled women and for more disabled women this was not possible at all [2].

This type of obstetric violence has a long-lasting influence on patients’ willingness to use health services and seeking for obstetric care. Withdrawal from healthcare services not only applies to obstetric care, but to all other forms of medical services. If stigma and discrimination occur during labor and delivery among disabled women, they may perceive a health facility not for disabled women, but it is for nondisabled women. This in turn may be discouraged to seek any type of facility care and potentially jeopardizing the safety of their pregnancy outcome in subsequent pregnancies [34–36].

Because their right to privacy is undervalued or not valued at all, women with disabilities may be subjected to prolonged periods of physical discomfort or embarrassment. In the current study, the majority of disabled women's childbirths at the facility were exposed for all to see, and the care providers allowed them to be necked. The windows in the laboring room were broken, and the delivery room doors were left wide open for anyone passing by to observe a woman in labor. In this study, disabled mothers also experienced their personal issues were being discussed with the medical team. Women’s privacy intentionally or unintentionally violated in the current study. This study parallels the explanations in Nigeria and Iceland [7, 29].

The lack of privacy has real consequences for birth experiences for such types of women, as seen labour rooms with collective accommodating up to four women, whose beds are separated by drapes or not separated at all. This may lead to perceived loss of confidential care at health facilities. Therefore, maternity wards should at least be secured, individual sensitive and suitable for disabled women for privacy for the sake of mother’s confidence to facility birth because the health facility should at least be a better place for the maternity service than women’s home [37].

The two major types of neglect and abandonment experienced by disabled women in the study area were a lack of access to timely healthcare and a loss of trust in facility care. Disabled women were left alone, struggling to survive alone, losing hope at the hands of healthcare providers, and locked in filthy toilets with no medical assistance from healthcare providers. It's a nightmare scenario for a blind woman and her baby to be on the toilet without any postnatal care. The consequences of being locked in a toilet could be multifaceted: it could harm the health of a newborn born from the sterile environment of his or her womb; it could be a serious violation of human rights by leaving a helpless mother and her baby to struggle alone in an unsafe environment; and it could cause psychosocial and physical harm.

As previously stated, they have also cursed the day they gave birth at that health facility where laboring women are left alone with no assistance. Similar experiences have been reported of a lack of the right to timely care and a loss of trust in facility care [38] It is frightening to be left alone or to miss timely obstetric care, especially for women who are handicapped. As a result, they lost faith in healthcare providers. An obstetric care seeking woman who went to the facility should not be left alone to suffer without assistance; otherwise, her visit to the health facility will be futile.

The clinical implications of this finding could be significant regarding ensuring quality, equal, individualized care. Women with disabilities have been victims of obstetric violence, which has a significant impact on questioning institutional delivery preferences, which may result in adverse maternal, fetal, and neonatal outcomes due to complications associated with home delivery. On top of that, women in the recent study subjected to different forms of obstetric violence had a deteriorative effect on the health of their newborns, particularly long-term mental injury and morbidity and mortality of newborns due to lack of or insufficient postnatal care as they were left locked in disgusting toilets, which are very infectious and never for postnatal stay.

Furthermore, the discovery has implications for human rights. Obstetric violence intersects with multiple human rights violations (intersectional reproductive health harm) because it encompasses a number of human rights, including the rights to health, privacy, freedom from discrimination, freedom from violence, and freedom from torture and other ill-treatment, among others. And it is lost on both counts or overlap multiple problems for a disabled woman.

## Limitations of the study

There are numerous limitations to our research. It was carried out with a small sample size and over a small area, making it difficult to generalize the results. However, given that we explored obstetric violence among disabled women in both urban and rural settings, it may still provide useful evidence to field researchers. It may also provide a finding for similar research in Ethiopia and other countries.

Furthermore, because this is a qualitative study, there may be limited generalizability outside of our context. An additional limitation is that participants in the current study may regard some abusive care as normal obstetric care. As a result, some aspects of obstetric violence may be missed. Limited literature in the field of study may also limit our study’s discussion section and the data collection methods are all in-depth interviews, which means that all information was gathered through in-depth interviews. The study's strength, however, may be in addressing the invisible wound among forgotten women.

## Conclusions and recommendations

The service quality was appalling, with reports of obstetric violence among this vulnerable group of women imposing a double burden on them (overlap of disability and obstetric violence). Despite the World Health Organization's recognition of the need for high-quality perinatal care and equitable access to maternity services for every woman, disabled women were subjected to various forms of obstetric violence. According to the findings, there is a need to improve maternity care for disabled women by implementing comprehensive, culturally sensitive, client-sensitive special services and providing sensitivity training to healthcare providers, ensuring satisfied, equitable, and quality obstetric care for every woman despite of their physical, sociodemographic and socioeconomic variations. Creating a rights-based policy that prioritizes disabled women and addresses obstetric violence requires the development of a broad-based provision for the elimination of obstetric violence in maternity care settings. Obstetric violence should be a top priority for every health-care provider. In particular, WHO must ensure that the situation of disabled women is recognized as a special issue, and that obstetric violence is recognized as a health and human rights violation. We also recommend a mixed approach to quantifying obstetric violence and an observatory method of data collection for further researchers.

## Data Availability

The datasets generated and /or analyzed during the current study are not publicly available due to preserving participant anonymity but are available from the corresponding author upon request through the email address (are.wud16@gmail.com).
